# Concomitant treatment of brain metastasis with Whole Brain Radiotherapy [WBRT] and Temozolomide [TMZ] is active and improves Quality of Life

**DOI:** 10.1186/1471-2407-7-18

**Published:** 2007-01-25

**Authors:** Raffaele Addeo, Michele Caraglia, Vincenzo Faiola, Elena Capasso, Bruno Vincenzi, Liliana Montella, Rosario Guarrasi, Luigi Caserta, Salvatore Del Prete

**Affiliations:** 1 Oncology Department, "S. Giovanni di Dio" Hospital, ASL Napoli3 Via Giovanni XXIII 80028 Frattaminore, Naples, Italy; 2 Experimental Pharmacology Unit, National Institute of Tumours "Fondaz. G. Pascale" of Naples, Italy; 3 Section of Oncology Campus Biomedico University; Rome, Italy; 4 Senology Unit, Distretto 65 ASL Napoli^3^, Arzano, Naples, Italy

## Abstract

**Background:**

Brain metastases (BM) represent one of the most frequent complications related to cancer, and their treatment continues to evolve. We have evaluated the activity, toxicity and the impact on Quality of Life (QoL) of a concomitant treatment with whole brain radiotherapy (WBRT) and Temozolomide (TMZ) in patients with brain metastases from solid tumors in a prospective Simon two stage study.

**Methods:**

Fifty-nine patients were enrolled and received 30 Gy WBRT with concomitant TMZ (75 mg/m2/day) for ten days, and subsequently TMZ (150 mg/m2/day) for up to six cycles. The primary end points were clinical symptoms and radiologic response.

**Results:**

Five patients had a complete response, 21 patients had a partial response, while 18 patients had stable disease. The overall response rate (45%) exceeded the target activity per study design. The median time to progression was 9 months. Median overall survival was 13 months. The most frequent toxicities included grade 3 neutropenia (15%) and anemia (13%), and only one patient developed a grade 4 thrombocytopenia. Age, Karnofsky performance status, presence of extracranial metastases and the recursive partitioning analysis (RPA) were found to be predictive factors for response in patients. Overall survival (OS) and progression-free survival (PFS) were dependent on age and on the RPA class.

**Conclusion:**

We conclude that this treatment is well tolerated, with an encouraging objective response rate, and a significant improvement in quality of life (p < 0.0001) demonstrated by FACT-G analysis. All patients answered the questionnaires and described themselves as 'independent' and able to act on their own initiatives. Our study found a high level of satisfaction for QoL, this provides useful information to share with patients in discussions regarding chemotherapy treatment of these lesions.

## Background

Brain metastases [BM] represent an important cause of morbidity and mortality in cancer patients, and are the most common intracranial tumor, occurring in approximately 10% to 30% of adult patients with cancer [1]. The risk of developing brain metastasis varies according to primary tumor type. Approximately half of all brain metastases occur due to lung cancer. More than 80% of brain metastases are detected after the primary tumor has been diagnosed; less frequently brain metastasis represent the first manifestation of neoplasia and/or are diagnosed at the same time as the primary tumor. The incidence of these metastases has increased in recent years for several reasons and they are associated with poor prognosis. The median survival time of untreated patients is approximately 1 month [1]. Often these patients have severe neurologic symptoms with a decrease in survival and quality of life.

Treatment choices are limited: only patients with a single brain metastasis benefit from surgery or radiosurgery. Frequently the palliative approaches focused on symptomatic care remain the standard treatment to relieve neurologic symptoms, primarily with the use of corticosteroids and anti-convulsant [2]. However, single metastases are rare and whole brain radiotherapy remains the standard treatment for most [3]. WBRT improves specific neurologic symptoms in the majority of patients [4], but response duration is short and the treatment may be associated with late complications. Phase III trials of the Radiation Therapy Oncology Group (RTOG) showed that treatment of brain metastasis with WBRT results in a median survival of 4 to 6 months and improves the neurologic function in most patients. No difference in median survival or 1-year survival has been seen between various dose groups, including a comparison between standard fractionation (30 Gy in 10 daily fractions) and accelerated hyperfractionation (1.6 Gy twice daily to 54.4 Gy) [5].

Patients with brain metastases represent a heterogeneous population. The Radiation Therapy Oncology Group classification derived from a recursive partitioning analysis (RPA) identified three groups of patients according to prognostic factors related to tumour based on Karnofsky performance score, primary tumor status, presence of extra-cranial metastases and age. Patients with KPS ≥70, age < 65 years, no extra-cranial metastases and controlled primary tumor are considered Class I and have a median survival of 7.1 months; patients with KPS< 70 are class III with a median survival of 2.3 months. All other patients belong to class II with a median survival of 4.2 months [6]. Most patients are in class II and III and the WBRT remain standard treatment.

The role of systemic chemotherapy in the management of BM is limited and controversial. The limited ability of most chemotherapeutic agents to cross the blood-brain barrier is believed to be one of the principal reasons these agents are less active against disease in the central nervous system than against extra-cranial, systemic disease [7]. Importantly, most patients have received most of the standard chemotherapy agents by the time they develop brain metastases and this increases tumor resistance. In newly diagnosed brain metastases, the tumors are responsive as the primary systemic cancer, as demonstrated by several phase II studies. The response rates ranging from 50% with 80% in patients with primary breast and lung cancer reported in some studies are associated to severe adverse events [8]. Results of a few phase III studies comparing chemotherapy alone with combined chemotherapy and WBRT do not allow firm conclusions [9], and studies comparing chemotherapy alone to WBRT alone are lacking. Nevertheless, it appears reasonable to consider chemotherapy for brain metastases in specific situations, such as chemosensitive primary tumor, or systemic metastases requiring chemotherapy. In fact, most patients are in class II and III and WBRT remains standard treatment.

Temozolomide (TMZ), an oral imidazotetrazinone methylating agent has demonstrated schedule-dependent activity in several cancers, including gliomas [10]. TMZ is rapidly absorbed and is spontaneously cleaved in vivo to monoethyl triazenoimidazole carboxamide (MTIC), a reactive DNA methylating species. The cytotoxicity of MTIC is thought to be caused by methylation of the O^6 ^position of guanine [11]. TMZ is highly bio-available after oral administration, has excellent central nervous system penetration, and reaches the brain in therapeutic concentrations [12]. Myelosuppression is the primary toxicity associated with TMZ, but it is non-cumulative, and manageable in the majority of patients.

We have previously reported the results of a phase II study demonstrating that the temozolomide/pegylated doxorubicin regimen was well tolerated with encouraging activity in brain metastases from solid tumors [13]. Previous studies have reported on the use of TMZ in the setting of recurrent or progressive brain metastasis with modest results [14,15]. The concomitant use of TMZ and WBRT is well tolerated and in a randomised phase II trial, showed a significantly higher response rate in patients receiving TMZ and radiotherapy versus radiotherapy alone [16,17], confirming the in vitro data on the combination of temozolomide with radiation [18].

The primary aim of this study was to confirm the efficacy and safety of the combination of TMZ and WBRT in patients with previously untreated BM from solid tumours. The primary end points were conventional clinical symptoms evaluation and neuro-imaging response criteria. We also evaluated the impact of this treatment combination on the quality of life of the patients enrolled in the study, overall survival, progression free survival, safety and tolerability.

## Methods

### Patient eligibility criteria

Adult patients with histologically proven primary cancer with measurable brain metastases assessable by contrast-enhanced computed tomography (CT) scan or gadolinium-enhanced magnetic resonance imaging (MRI) that were not suitable for surgery or radiosurgery, were eligible for the study. Eligibility criteria included age ≥18 years; KPS ≥50; a life expectancy of ≥3 months; serum creatinine and total serum bilirubin ≤1.5 times the upper limit of normal; adequate hematologic and hepatic function (including absolute neutrophil count ≥1500/mm^3^, platelet count 100000/mm^3^, AST and ALT ≤3 times the upper limit of normal). Patients who had received prior treatment for brain metastases, or those with severe inter-current medical illness or symptomatic heart disease, or pregnant or lactating women, were ineligible. Eligible patients were required to have fully recovered from previous therapy. The Institutional Ethics Committee approved the protocol and patients were required to give informed consent before beginning the treatment.

### Study design

A two-stage Simon accrual design was adopted for this phase II trial [19]. The minimum target activity level was 20% and early discontinuation of the study was planned in case of no response in the first 12 assessable patients. Alternatively, a planned sample size of 55 evaluable patients was chosen to better estimate efficacy; with 25% maximum width of the 95% confidence interval (CI) for an expected 30% overall response rate. Time to progression (TTP) was measured from the date of registration to the date of documented progressive disease or death. Overall survival was measured from the time of registration to the date of death resulting from any cause. The primary endpoints were the analysis of tumor response and feasibility. Secondary endpoints were the evaluation of TTP, overall survival (OS), and median time to response. TTP was measured from the date of registration to the date of documented progressive disease or death. OS was assessed from the time of registration to the date of death resulting from any cause. TTP and OS were both determined by Kaplan-Meier product limit method [20]. The median response duration was calculated from the date of registration to the date of disease progression or death. All analyses were performed following an intention to treat approach.

### Treatment schedule

Planned conventional WBRT was administrated with a daily dose of 3 Gy × 5 days each week for two weeks to a total dose of 30 Gy. TMZ was administrated orally at a dosage of 75 mg/m2/day during the radiation treatment and 150 mg/m2/day × 5 days every 28 days after RT to fasting patients for a maximum of six additional cycles. Treatment was continued until disease progression or unacceptable toxicity. All patients received corticosteroids at the lowest dose necessary to maintain neurologic stability, and anti-convulsivants were given when indicated. Blood counts were assessed weekly during therapy; liver, renal function tests and electrolytes were monitored before each cycle.

### Patient evaluation

Baseline assessment were performed within 1 week before the initiation of radiation treatment and included a complete history and physical and neurologic examination, evaluation of KPS, complete blood cell count, creatinine clearance, liver enzymes, total bilirubin, and serum electrolytes, total protein, albumin, and calcium. All patients underwent weekly physical and neurologic examinations during concurrent treatment and a complete clinical evaluation, laboratory tests, KPS and CT or MRI 30 days after WBRT. Laboratory tests were done before each TMZ cycle.

Target lesions were assessed by computed tomography (CT) or gadolinium-enhanced magnetic resonance imaging (Gd-MRI) in the 2 weeks before the onset of treatment, all scans were centrally reviewed by two blinded radiologists. Radiologic evaluation of target lesions was performed every time clinical evidence of neurologic progression was noted or every two 28-day cycles (within one week before the day of dosing), and 4 weeks after the least treatment cycle, according to the WHO/ECOG criteria [21] as described below.

Complete response (CR): disappearance of all known brain metastases. Partial response (PR): 50% or greater decrease in measurable brain lesions or an objective improvement in evaluable brain lesions. Stable disease (SD): brain lesions unchanged (< 50% decrease or < 25% increase in the size of measurable lesions). Progressive disease (PD): >25% increase in size of some or all of brain lesions and/or the appearance of any new brain lesions. No CR, PR, or SD before increased disease or new lesions. No brain lesion regresses.

All adverse events were recorded and graded according to the CTC-NCI criteria (version 2.0).

### Evaluation of QoL

Health-related QoL was measured using subject-completed Functional Assessment of Cancer Therapy (FACT) and FACT- brain metastases questionnaires developed for use in clinical trials. The FACT-General (FACT-G) Version 4 is a 27-item core multidimensional questionnaire evaluating various domains of QL including physical, functional, family, social, and emotional domains. Items are summed to give scores for each domain, and an overall QL score. The FACT-G questionnaire is available in 24 different languages and is widely accepted in the Italian language [22]. The FACT-Br scale contains 53 questions with high validity and good psychometric properties and efficacy to assess QoL in patients with brain tumors [23]. We selected only 26 of what there were considered the most relevant questions. Patients responded to QoL questions on a five-point Likert-type scale ranging from '0' ('not at all') to '4' ('very much') [24].

The questionnaires were used to evaluate the QoL at baseline, after 3 months, 6 months and 9 months.

### Statistical analysis

A univariate analysis for each prognostic variable on overall survival and progression free survival was estimated according to the Kaplan-Meier method [20]. The terminal event was death attributable to any cause. The statistical significance of the differences in survival distribution among the prognostic groups was evaluated by the log-rank test. Factors reaching significance of univariate analysis were entered in a multivariate analysis using the Cox stepwise logistic regression test to investigate the independence of the various risk factors.

P values <0.05 was regarded as statistically significant in two tailed tests. The QoL data were analysed by ANOVA test. SPSS software (version 10.00, SPSS, Chicago) was used for statistical analysis.

## Results

### Patient characteristics

Between October 2001 and June 2004, 63 patients were enrolled, and 59 patients completed RT and were assessable for efficacy and safety. Four patients refused treatment. The demographics and baseline disease characteristics of the assessable patients are listed in Table 1.

Among 59 assessable patients 22 (37%) had non-small-cell lung cancer, 16 (28%) had other cancer (colo-rectal carcinomas, melanoma, ovarian cancer and testicular cancer), and 21 (35%) had breast cancer; 33 (56%) of 59 patients had metastases in other organs. The majority of patients, 34 out 59, had received chemotherapy for primary cancer before entering the study. According to RPA analysis, class I included 21 patients, the class II 22, class III 16 patients.

### Response of Brain Lesions to Treatment

**Figure 1:**
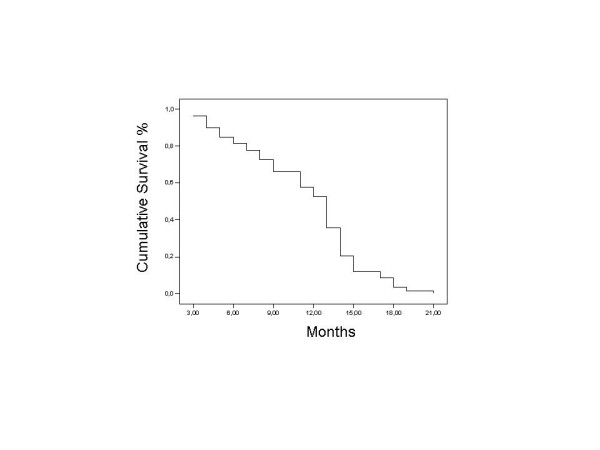
The Overall survival has been constructed by the Kaplan-Meier method. The median OS was 13.00 months (95% C.I.: 11.89 – 14.11).

Fifty-nine patients were assessable for response. Five CRs, in patients with breast cancer and NSCLC, and 21 PRs were recorded in 12/21 patients with breast cancer, 8/22 patients with NSCLC and 1 in other cancers, while a stable disease was achieved in other 19 patients. All the responses have been defined on the basis of measurable brain lesions. As shown in Table 2, the overall response rate was 44% (C.I. 38.3–56.6%), while the disease control rate was 76.3% (C.I. 61.2–81.9%). The median progression free survival was 9.00 months (95% CI: 7.93 – 10.07). Patients treated with WBRT and TMZ had a slight improvement in OS: 13.00 months (95% C.I.: 11.89 – 14.11) compared to data in the literature for WBRT alone (Fig. 1).

The statistical analysis of factors predictive for response revealed that the BM disease control, after WBRT/TMZ treatment, depended on age ≤ 65 years (p < 0.0001), Karnofsky Performance Status ≥ 70 (p < 0.0001), no extra-cranial disease (p = 0,012), RPA class I (p < 0.0001) (Table 3).

Univariate analysis for PFS showed statistically significant differences for the presence of extra-cranial disease (p = 0.02), age < 65 years, KPS ≥ 70 and RPA class I (Table 4). Factors predictive for improved overall survival, in univariate analysis, included KPS ≥ 70, age ≤ 65 years and lower RPA class (Table 4).

To confirm these results we performed a multivariate analysis: the patients with KPS ≥70 (p < 0.05) and those included in the first RPA class (p = 0.001), respectively, showed a statistically significant improvements of the time to progression (Table 5). The multivariate analysis for OS confirmed the central role for KPS ≥70 (p = 0.024) and the first RPA class (p = 0.004) in predicting OS (Table 5). The multivariate analysis performed excluding RPA confirmed the role of all radiological response, age and age in predicting both OS and PFS (Table 6).

### Safety and Tolerability

Acute side-effects are summarized in Table 7. The addition of TMZ to RT was generally well tolerated. The most common toxicities were neutropenia (35%) and anemia (22%). Grade III neutropenia developed in 9 patients (15%). The most frequent non hematologic events were ≥ grade 2 occurring in about 5% of patients including nausea, vomiting, headache, fatigue and rash. Most side effects were grade 2 and were well controlled by supportive care. Hepatic, renal, cardiac or severe neurological toxicity were not observed in our series. One patient required dose reduction of TMZ (125 mg/m^2^/day) as a result of grade IV thrombocytopenia, and four patients had a dose delay because of TMZ-associated persistent neutropenia.

The WBRT/TMZ combined treatment was overall well tolerated in almost all patients.

### Impact of WBRT/TMZ on quality of life

**Figure 2:**
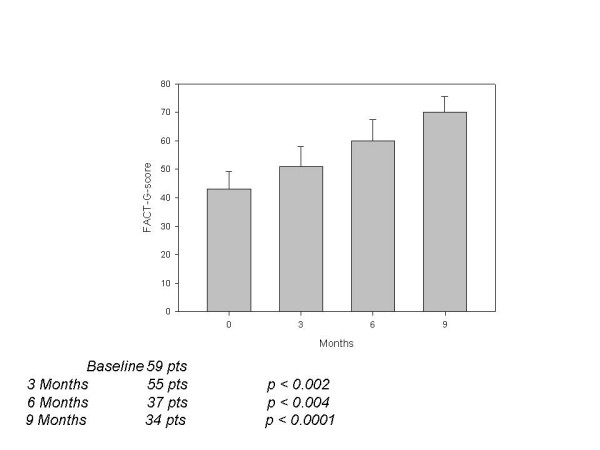
Variations (mean ± SD) in QoL in TMZ/WBRT treated pts. Statistical analysis has been performed by ANOVA comparing different time pts to the baseline values.

All patients who answered the questionnaire at baseline were included in the evaluation, and the FACT-G score was compared with the baseline value for each of these patients. Baseline questionnaires were completed by 59 patients (100%). The rate of completed questionnaires decreased as follows: first evaluation, after 3 months, 93%, second evaluation, after 6 months, 62 %, and third evaluation, after nine months, 57%. As shown in Fig. 2, we found a statistically significant improvement measured by the questionnaire. These results evidence an interesting positive impact of this therapy in patients with brain metastases after 3 and 6 months from the beginning of the treatment with TMZ. These data suggest that TMZ has superior palliative effects despite the presence of toxicity. Overall, the aspects of the quality of life that are assessed with the FACT-G questionnaire were maintained or improved during treatment, with the greatest benefit occurring for BM-specific concerns. The FACT-Br questionnaire confirmed these encouraging results as we found an amelioration of quality of their life after three months. From the FACT-Br survey, 26 questions were asked and the mean and median scores for each question from all the respondents were tabulated. The analysis of the respondents, 34 patients, reported that they were 'quite a bit' or 'very much' content with the quality of their life after 3 months of treatment (79% positive respondents to 21% negative respondent), on the other and, the baseline value were following: positive respondents 51%, 21 patients, and negative respondents 49% (those patients who responded "not at all"/"a little bit"), 20 patients. The completion rates for the FACT-Br scores at 6 (38%) and 9 (30%) months were too low to provide a meaningful comparison.

## Discussion and Conclusion

The clinical studies evaluating the role of chemotherapy in BM have been mostly non randomised Phase II trials. The results have been often divergent due to variability in several factors, including tumor histology, presenting stage, previous use of chemotherapy, etc. [25]. Two randomized phase III studies have confirmed the efficacy of chemotherapy in BM. One, of concurrent chemotherapy plus radiotherapy in BM from non-small-cell lung cancer [26], suggested that the timing of WBRT, with respect to chemotherapy with cisplatin and vinorelbine did not influence survival. The other, evaluating effect of adding WBRT to teniposide in small cell lung cancer, resulted in a better response rate and longer time to BM progression but had no impact on survival [27]. Several trials of TMZ plus radiation therapy have been conducted in patients with newly diagnosed brain metastases. Previous studies demonstrated that TMZ treatment is safe and well-tolerated when combined with RT but no significant improvements in survival was noted, compared to WBRT alone. Improvement in neurologic functional status has been reported with the combination, but there are no significant data about the influence on overall responses and QoL. In fact, the efficacy and safety of temozolamide concurrently with WBRT for patients with newly diagnosed brain metastases was evaluated in recent trials. The largest of these trials demonstrated a significant improvement of response rate and trend toward improved survival in the arm of combined therapy (8.3 vs. 6.3 months; p = 0.179) that was however not statistically significant [7]. Our data, revealing a 44% overall response rate (CR+PR) with 8,5% CR rate and the 76% brain disease control rate, appear encouraging. Patients treated with WBRT and TMZ had a slight improvement in OS as compared to the study of Antonadou et al. that yielded a median OS time of 8.6 months in the arm of concomitant treatment with WBRT and temozolamide [16]. More recently, a phase II trial of temozolomide and cisplatin followed by whole brain radiotherapy in NSCLC patients with brain metastases demonstrated a median OS of 5 months [28].

The responses were independent from the type of the primary tumor, gender and previous chemotherapy. In details, also the inclusion of brain metastases from breast cancer in the analysis did not change these results (data not shown). Furthermore, in our series KPS <70, age >65 years, presence of other extra-cranial metastases and RPA class III represent unfavourable factors for response. We have also demonstrated that KPS and age influence PFS and OS.

The results of this study suggest that the combination of WBRT and TMZ has a significant clinical activity in patients with brain metastases from solid tumours. The overall objective response plus stabilization rate of 78,9% is similar to that typically achieved with WBRT alone (61–91%). However, the responses achieved in this study were also reasonably durable with a median progression free survival of 9 months. Moreover, the median survival of 13 months is good for such an advanced-staged patient population. These results appear better than others obtained in previous trials; a possible explanation is the presence in our series of a high number of patients in the I and II RPA class.

This regimen was generally well tolerated by almost all patients, including elderly patients (>70 years) (12/59). The addition of daily TMZ to WBRT resulted in only one grade 4 hematologic toxicity. When neutropenia or thrombocytopenia developed, it resolved quickly and resulted in only minor treatment delays up to one week, with only one patient requiring dose reduction to 125 mg/m^2 ^of TMZ. In our series of patients we observed a considerable improvement in QoL, measured using the FACT-G and FACT-Br questionnaires. These data confirm previous results which showed an amelioration of QoL also in patients who did not obtain an objective response.

The results of our QoL analysis shows a high level of satisfaction among patients who have undergone TMZ plus WBRT treatment for brain metastases and provides excellent support for its acceptability. The benefits of treatment-related efficacy were not affected by symptoms caused by the toxic effect. The results of the present study support the efficacy and safety of WBRT and TMZ in the treatment of patients with BM from a variety of solid tumors and confirm that this combination can also offer significant palliation for BM, improving global QoL. Though the presented data looks promising, this will need to be further validated within the settings of a randomized trial.

## Abbreviations

Whole brain radiotherapy (WBRT), Temozolomide (TMZ), Brain metastases (BM), Quality of life (QoL)

## Competing interests

The author(s) declare that they do not have competing interests.

## Authors' contributions

RA and SDP projected the study and participated in its design and coordination;

RA and MC participated in drafting the manuscript.

RG, VF, LC and LM carried out all the treatment and the patients' follow-up.

LM, RA and SDP participated in acquisition of data and gave substantive intellectual contribution when revising it critically;

BV and EC performed the analysis of the data.

All authors read and approved the final manuscript.

## Pre-publication history

The pre-publication history for this paper can be accessed here:



## Figures and Tables

**Table 1:** Patients characteristics

**Characteristics**	**No. of patients**
Age (y)	
≥*65*	*27*
<*65*	*32*
Gender	
*Male*	*27*
*Female*	*32*
KPS	
≥*70*	*40*
<*70*	*19*
Primary tumour	
*Lung*	*22*
*Breast*	*21*
*Other*	*16*
Extracranial metastasis	
*Yes*	*33*
*No*	*26*
RPA Class	
*I*	*21*
*II*	*22*
*II*	*16*
Previous chemotherapy	
*Yes*	*34*
*No*	*25*

**Table 2:** Brain Lesion Response to Treatment

	TMZ/WBRT	
Parameter	No. pts	%
Complete Response [CR]	5	8.5
Partial Response [PR]	21	35.5
Objective Response [CR+PR]	26	44
Stable Disease	19	32.3
Progressive Disease	14	23.7

**Table 3:** Analysis of clinical predictive factors.

**Clinical predictive factors for response**			
	Tumor control (SD, CR, PR)	Brain progression (PD)	P value

Lung Cancer	16	7	0.363
All the others	29	7	
Males	25	7	0.321
Females	17	10	
KPS <70	6	10	**< 0.0001**
KPS ≥70	29	4	
No previous chemotherapy	20	5	0.758
Previous chemotherapy	25	9	
No extracranial disease	25	2	**0.012**
Extracranial disease	20	12	
Age > 65 years	6	10	**<0.0001**
Age ≤65 years	39	4	

**RPA as a predictive factor for response**			

	Tumor control (SD, CR, PR)	Brain progression (PD)	P value

RPA 1	14	0	**< 0.0001**
RPA 2	25	4	
RPA 3	6	10	

**Table 4:** Statistical analysis for OS and PFS.

**Univariate Analysis for Overall Survival (OS) cancer patients with brain metastases**			
	**OS (months)**	**95% C.I.**	**P value**

Radiological response:			
Tumor control (SD, CR, PR)	13.00	12.35 – 13.65	**< 0.0001**
Brain progression (PD)	5.00	3.83 – 6.17	
Lung Cancer	13.00	12.10 – 13.90	0.9263
All the others	12.00	10.32 – 13.68	
Males	11.00	7.63 – 14.37	0.239
Females	13.00	12.22 – 13.78	
KPS <70	5.00	2.39 – 7.61	**0.0006**
KPS ≥ 70	13.00	12.37 – 13.63	
No previous chemotherapy	13.00	11.53 – 14.47	0.7568
Previous chemotherapy	12.00	10.86 – 13.14	
No extracranial disease	13.00	13.19 – 14.81	0.0681
Extracranial disease	10.00	7.31 – 14.69	
Age > 65 years	6.00	2.56 – 9.23	**0.0050**
Age ≤ 65 years	12.00	10.01 – 13.67	
RPA 1	13.00	11.95 – 14.05	**0.0025**
RPA 2	13.00	12.16 – 13.84	
RPA 3	5.00	2.39 – 7.61	

**Univariate Analysis for Progression Free Survival (PFS) cancer patients with brain metastases**			

	**PFS (months)**	**95% C.I.**	**P value**

Radiological response:			
Tumor control (SD, CR, PR)	10.00	8.54 – 11.46	**< 0.0001**
Brain progression (PD)	4.00	3.63 – 4.37	
Lung Cancer	9.00	7.85 – 10.15	0.3578
All the others	10.00	7.67 – 12,33	
Males	9.00	7.42 – 10.58	0.4815
Females	9.00	6.50 – 11.50	
KPS <70	4.00	3.22 – 4.78	**0.0016**
KPS ≥ 70	10.00	8.40 – 11.60	
No previous chemotherapy	10.00	8.05 – 11.95	0.7835
Previous chemotherapy	8.00	6.73 – 9.27	
No extracranial disease	11.00	9.54 – 12.45	**0.0247**
Extracranial disease	8.00	5.80 – 10.20	
Age > 65 years	5.00	4.10 – 6.43	**0.02**
Age ≤ 65 years	10.00	7.22 – 11.98	
RPA 1	11.00	8.56 – 13.44	**0.0027**
RPA 2	9.00	7.25 – 10.75	
RPA 3	4.00	3.22 – 4.78	

**Table 5:** Multivariate analysis for OS and PFS.

**Overall Survival (OS)**	**P value**
Tumour control (SD, CR, PR)	**0.007**
Brain progression (PD)	
KPS <70	**0.024**
KPS ≥ 70	
Age ≤ 65 years	0.792
Age > 65 years	
RPA 1	**0.004**
RPA 2	
RPA 3	

**Progression Free Survival (PFS)**	**P value**

Tumor control (SD, CR, PR)	**0.001**
Brain progression (PD)	
KPS <70	**0.05**
KPS ≥ 70	
No extracranial disease	0.810
Extracranial disease	
Age > 65 years	0.310
Age ≤ 65 years	
RPA 1	**0.001**
RPA 2	
RPA 3	

**Table 6:** Multivariate analysis for OS and PFS without RPA.

**Overall Survival (OS)**	**P value**
Tumour control (SD, CR, PR)	**<0.0001**
Brain progression (PD)	
KPS ≤60	**0.002**
KPS >60	
Age ≤65 years	**0.01**
Age >65 years	

**Progression Free Survival (PFS)**	**P value**

Tumor control (SD, CR, PR)	**<0.0001**
Brain progression (PD)	
KPS ≤60	**0.003**
KPS >60	
No extracranial disease	0.325
Extracranial disease	
Age >65 years	**0.01**
Age ≤65 years	

**Table 7:** Most common and serious Adverse events During TMZ/WBRT treatment according to Common Toxicity Criteria extended by National Cancer Institute, (NCI CTC) in the 59 enrolled patients.

	Grade 2		Grade 3		Grade 4	
**Toxicity**	No. pts	%	No. pts	%	No. pts	%

Neutropenia	15	25	9	15	-	-
Thrombocytopenia	4	7	2	3	1	1
Anemia	7	12	8	13	-	-
Alopecia	2	3	-	-	-	-
Vomiting	6	10	2	3		
hand and foot syndrome	2	3	-	-	-	-
Headache	5	8	1	1	-	-
Nausea	5	8	2	3	-	-
Tremor	4	7	2	3	-	-
Nerosensory	3	5	-	-	-	-
Fatigue	7	12	2	3	-	-
Rash	8	13	3	5	-	-
